# Extrahepatic Bile Duct Organoids as a Model to Study Ischemia/Reperfusion Injury During Liver Transplantation

**DOI:** 10.3389/ti.2024.13212

**Published:** 2024-09-11

**Authors:** P. Kreiner, E. Eggenhofer, L. Schneider, C. Rejas, M. Goetz, N. Bogovic, S. M. Brunner, K. Evert, H. J. Schlitt, E. K. Geissler, H. Junger

**Affiliations:** ^1^ Department of Surgery, University Hospital Regensburg, Regensburg, Germany; ^2^ Department of Pathology, University Hospital Regensburg, Regensburg, Germany

**Keywords:** regenerative medicine, organoids, liver transplant, cholangiopathy, cholangiocyte organoids

## Abstract

Biliary complications are still a major cause for morbidity and mortality after liver transplantation (LT). Ischemia/reperfusion injury (IRI) leads to disruption of the biliary epithelium. We introduce a novel model to study the effect of IRI on human cholangiocytes using extrahepatic cholangiocyte organoids (ECOs). Extrahepatic bile duct tissue was collected during LT at static cold storage and after reperfusion (n = 15); gallbladder tissue was used for controls (n = 5). ECOs (n = 9) were cultured from extrahepatic biliary tissue, with IRI induced in an atmosphere of 95% air (nitrogen), 1% O_2_ and 5% CO_2_for 48 h, followed by 24 h of reoxygenation. Qualitative and quantitative histology and qRT-PCR were performed to discern phenotype, markers of hypoxia, programmed cell death and proliferation. ECOs self-organized into circular structures resembling biliary architecture containing cholangiocytes that expressed EpCAM, CK19, LGR5 and SOX-9. After hypoxia, ECOs showed increased expression of VEGF A (*p* < 0.0001), SLC2A1 (*p* < 0.0001) and ACSL4 (*p* < 0.0001) to indicate response to hypoxic damage and subsequent programmed cell death. Increase in cyclin D1 (*p* < 0.0001) after reoxygenation indicated proliferative activity in ECOs. Therefore, ECO structure and response to IRI are comparable to that found *in-vivo*, providing a suitable model to study IRI of the bile duct *in-vitro*.

## Graphical Abstract

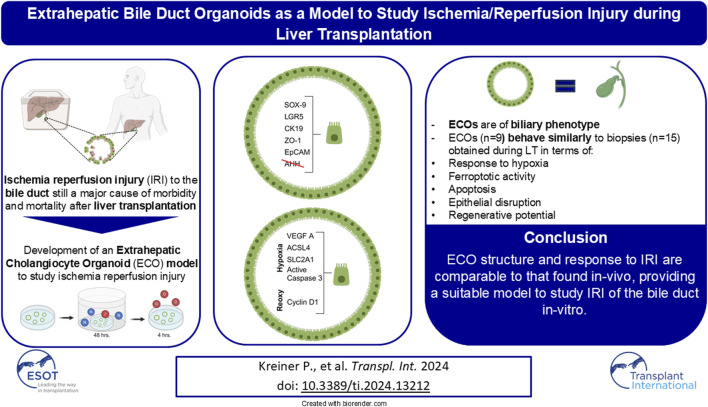

## Introduction

Post-transplant cholangiopathies commonly occur after liver transplantation (LT) [1–6]. Recent studies indicate a strong association between epithelial damage in the bile duct induced by IRI during LT and the development of post-transplant cholangiopathies [1, 7–10]. Newly developed technologies and methods in regenerative medicine and stem cell research potentially offer novel treatment options, such as the use of organoids to study and even treat cholangiopathies that arise after LT [11, 12]. Cholangiocyte organoids are a self-organized, three-dimensional tissue that mimics the key functional, structural and biological complexity of the biliary system [13]. Interestingly, previous research on cholangiocyte organoids has shown that while intrahepatic and extrahepatic cholangiocytes are initially morphologically different, they can remarkably change their phenotype according to their location [14]. Being able to culture various cholangiocytes as organoids provides a potentially useful tool to study bile duct biology in the setting of LT.

One of the main factors causing effects on liver bile ducts during transplantation is ischemia and reperfusion injury (IRI) that occurs during the organ retrieval and implantation process. Few cellular functions are unaffected, but classic markers of IRI include hypoxia-inducible factor 1-alpha (HIF-1α) and vascular endothelial growth factor A (VEGF A), which rise in response to tissue hypoxia and reperfusion [15, 16] HIF-1α is expressed ubiquitously in cells even under normoxic conditions, being ubiquitinated by von-Hippel-Lindau protein in the presence of oxygen [16]. VEGF A expression is linked to HIF-1α expression in response to hypoxia by molecular pathways [15, 16]. While HIF-1α is a transcription factor that exerts its effect within the cell [16], VEGF A is produced as a soluble growth factor that stimulates cells supporting vascular development and influences immune reactions to injury [17].

In terms of cell survival, ferroptosis is one pathway of programmed cell death that is a major contributor in IRI [18–21]. Ferroptosis is a highly conserved iron-dependent form of non-apoptotic cell death from an evolutionary standpoint [22], indicating its importance [23] During ferroptosis, iron-dependent lipid peroxidation occurs leading to loss of cell and mitochondrial membrane integrity [24], and subsequently to cell death Klicken oder tippen Sie hier, um Text einzugeben. Acyl-CoA long chain family member 4 (ACSL4) is critical for ferroptosis signaling, and therefore a specific biomarker of ferroptosis that we use in this study Klicken oder tippen Sie hier, um Text einzugeben.

The complex pathophysiological mechanisms induced by IRI in the biliary system during LT leading to post-transplant cholangiopathies remain poorly understood. To advance our knowledge it is necessary to develop *in-vitro* models that simulate IRI to bile ducts. In our current study we established an extrahepatic cholangiocyte organoid model mimicking IRI occurring during a liver transplant procedure. With this organoid model using *in-vitro* cultured extrahepatic cholangiocytes, we compare IRI biomarker responses to organoid injury with actual common bile duct specimens obtained at cold storage and after reperfusion during LT.

## Materials and Methods

### Study Setting

The study was approved by the University of Regensburg Ethics Committee (local ethics committee #16-101_5-101), using samples collected in the Department of Surgery. Written informed consent was given by all patients.

**FIGURE 1:**
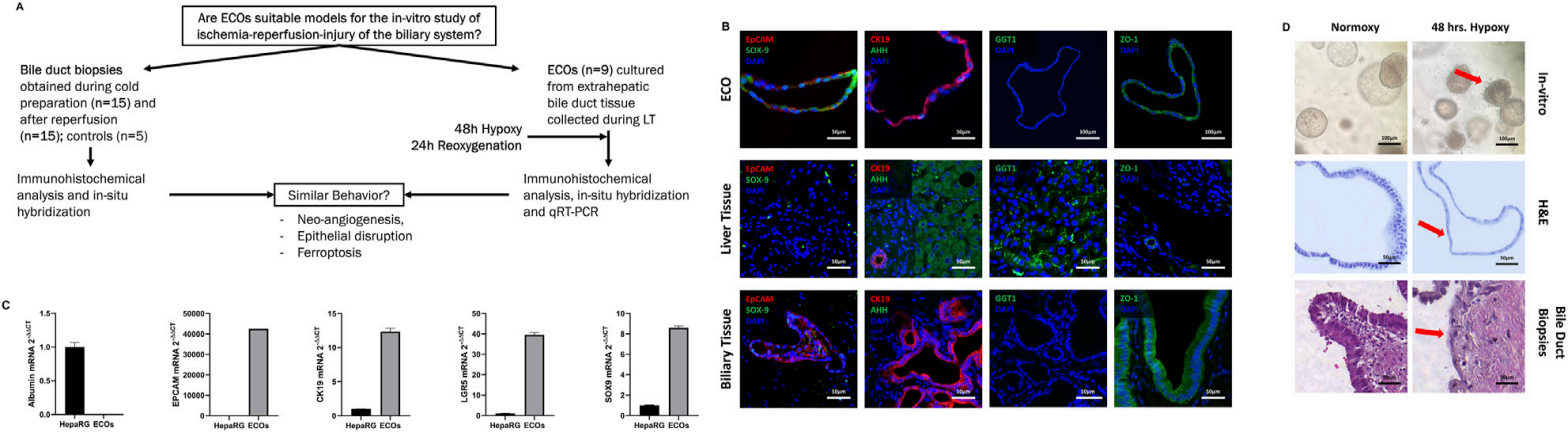
Study design and developing extrahepatic organoid model for Model to Study Ischemia/Reperfusion Injury during Liver Transplantation. **(A)** Research question and study design. **(B)** Representative multiplex immunohistochemistry of ECOs, healthy liver tissue and biliary tissue. ECOs as well as extrahepatic bile duct biopsies express key cholangiocyte markers: SOX-9, EpCAM (first image, top row and bottom row) and CK19 (second image, top row and bottom row). ECOs are negative for AHH (second image, top row) while AHH is expressed in liver tissue (second image, middle row). Furthermore, ECOs (fourth image, top row) as well as the human bile duct sample (fourth image, bottom row) expressed ZO-1. ZO-1 is not present in liver sample (fourth image, middle row). SOX-9, CK19and EpCAM is not expressed in hepatocytes of healthy liver biopsies (left picture, bottom row), while AHH stains hepatocytes in liver biopsies (second picture, middle row). CK19 can only be found in cholangiocytes of bile ducts in the Glisson triad of the liver sample (second picture, bottom row). GGT1 can be found in liver samples (third picture, middle row) but not in ECOs (third picture, top row) and bile duct biopsies (third picture, bottom row) respectively **(C)** qRT-PCR of ECOs was performed for key cholangiocyte markers: EpCAM, CK19, LGR5 and SOX-9. Cholangiocyte markers are highly expressed in ECOs and while not being expressed in the control group (hepatocyte: HEPA RG); albumin was highly expressed in HEPA RG cells, but not expressed in ECOs. **(D)** *Top Row:* ECOs in culture during normoxic (left) and hypoxia conditions (right). ECOs lose their shape and integrity when subjected to hypoxia, as indicated by a red arrow. *Middle Row:* H&E staining of ECOs during normoxic conditions (left; cylindric epithelium) and under hypoxia (right); there is visible flattening of epithelial cells during hypoxia, as indicated by a red arrow. *Bottom Row*: Biopsies obtain from undamaged bile duct (left, cylindric epithelium) and after ischemia and reperfusion (right), with visible disruption and flattening of the biliary epithelium (red arrow).

Bile duct biopsies were obtained (I) under static cold storage condition during back table preparation of donor livers at the recipient center and (II) approx. 1 h after reperfusion as well as controls from non-diseased cystic ducts obtained during cholecystectomy. These bile duct biopsies were then compared to ECOs subjected to hypoxia and reoxygenation in regard to their behavior regarding neo-angiogenesis (HIF-1α, VEGF A, SLC2A1), ferroptosis (ACSL4) and epithelial disruption to answer whether ECOs are a suitable model for the *in-vitro* study of ischemia-reperfusion-injury of the biliary system (Figure 1A). Control liver tissue biopsies were obtained after liver reperfusion.

### Specimen Collection

Human biliary tissue was collected during cholecystectomies of non-diseased gall bladders that were routinely performed in the scope of larger surgeries (n = 14). The control biopsies were taken from the gallbladder, before its blood supply were tampered with. We therefore achieved biopsies with no relevant ischemia time. Five specimens were formalin fixed and paraffin embedded (FFPE) and used as controls for immunohistochemical analysis. Nine specimens collected were used for ECO cultivation (n = 9). Furthermore, liver and bile duct biopsies were obtained during LT procedures. Attending transplant surgeons followed a detailed standardized sampling protocol to collect 2-mm-long circular specimens of common bile duct. Samples were taken at the beginning of the back-table procedure during transplantation surgery (n = 15), and after portal and arterial reperfusion prior to the biliary anastomosis (n = 15). A table with donor demographics is provided in the Supplementary Material (Supplementary Table S1).

### Organoid Cultivation

ECOs (n = 9) were initiated and cultivated from extrahepatic biliary tissue, as described by Sampaziotis et al. [11]. Briefly, intact extrahepatic biliary tissue was washed in Earle’s Balanced Salt Solution (EBSS; ref. #14155063, Gibco, Thermo Fischer Scientific, DE) and then finely minced using a disposable scalpel. Tissue fragments were digested into dispersed cells with 4 mL digestion solution (EBSS and collagenase (ref. # C9891, Sigma-Aldrich, Merck, DE)) at 37°C for 20 min. After filtering through a 70 µM CellStrainer, the cell suspension was washed twice in Advanced Dulbecco’s Modified Eagle Medium/Nutrient Mixture F-12 (ADMEM/F12; ref. #12634010, Gibco, Thermo Fischer Scientific, DE) containing HEPES buffer (ref. # H0887, Sigma-Aldrich, Merck, DE), L-glutamine (ref. #G7513, Gibco, Sigma-Aldrich, Merck, DE), and a mixture of antibacterial and antifungal agents (Anti-Anti; ref. #15240062, Gibco, Sigma-Aldrich, Merck, DE). Recovered cells were then suspended in Base Membrane Extract (BME; ref. #3533-010-02, R&D Systems, Bio-Techne, United States) and added to the culture plates. Supplemental start-up medium was added to the cultures consisting of nicotinamide (ref. #N0636, Sigma-Aldrich, Merck, DE), N-acetyl-L-cysteine (ref. #A9165, Sigma-Aldrich, Merck, DE), Y-27632 (ref. #1293823, biogems, United States), A 83-01 (ref. #9094360, biogems, United States), forskolin (ref. #1099, R&D Systems, Bio-Techne, United States), epidermal growth factor (ref. #AF-100-15, PeproTech, Thermo Fischer Scientific, DE), hepatocyte growth factor (ref. #100-39, PeproTech, Thermo Fischer Scientific, DE), fibroblast growth factor-10 (ref. #100-26, PeproTech, Thermo Fischer Scientific, DE), human [Leu^15^]-gastrin I (ref. #G9145, Sigma-Aldrich, Merck, DE), recombinant human noggin (ref. #120-10C, PeproTech, Thermo Fischer Scientific, DE), recombinant human R-spondin-1 (ref. #120-38, PeproTech, Thermo Fischer Scientific, DE), Wnt3A (ref. #H17001, Sigma-Aldrich, Merck, DE), hES cell cloning & recovery supplement (ref. #01-0014-500, Stemolecule, Reprocell, JAP), B-27 supplement (ref. #12587010, Gibco, Thermo Fischer Scientific, DE) and N-2 supplement (ref. #17502001, Gibco, Thermo Fischer Scientific, DE). Cells were incubated in a humidified incubator at 37°C, 5% CO_2_. After 3 days, the start-up medium was exchanged with expansion medium, which is start-up medium deprived of noggin, Y267632, Wnt3A and hES cell cloning & recovery supplement. ECOs were propagated by changing the expansion medium every 3 days, with regular splitting of the organoids to foster optimal conditions for ECO expansion.

### Hypoxia and Reoxygenation

To mimic IRI *in-vitro*, ECOs were subjected to hypoxia and reoxygenation according to established methodology [25–28]. For this, organoids were subjected to an atmosphere of 95% air (nitrogen), 1% O_2_ and 5% CO_2_ for 48 h to induce hypoxia. Following hypoxia, organoids were then re-oxygenated in the incubator at 21% O_2_, 5% CO_2_ and 37°C for another 24 h. Samples for both qRT-PCR and histology were collected after hypoxia induction and reoxygenation.

### Analysis

#### qRT-PCR

qRT-PCR was performed to answer whether ECOs expressed genes related to biliary phenotype. A HEPA RG cell line was used as control in determining biliary phenotype of ECOs. Furthermore, qRT-PCR was used to quantify markers of hypoxia, neo angiogenesis and ferroptosis in ECOs to show whether ECOs behave similarly *in-vitro* to human bile ducts during LT. Targets were chosen firstly with the intention of demonstrating that organoids cultivated from extrahepatic cholangiocytes were initiated correctly and retained their biliary phenotype. Therefore, epithelial cell adhesion molecule (EpCAM), SRY-box transcription factor 9 (SOX-9 [14]), leucine-rich repeat-containing G-protein coupled receptor 5 (LGR5) [11, 12], cytokeratin-19 (CK-19) [11, 12] and albumin mRNA were measured. Secondly, to assess whether hypoxia had been achieved, testing for HIF-1α [15], VEGF A [15, 16, 29], and glucose transporter 1 (SLC2A1) [16, 29–32] was performed. Thirdly, proliferative activity was measured using cyclin D1 [33]. Furthermore, to assess whether ECOs enter into apoptosis, qRT-PCR for regulators of mitochondrial membrane permeability BAX (pro-apoptotic) and Bcl-2 (anti-apoptotic) as well as Caspase 3 which is part of the execution pathway of both the intrinsic and the extrinsic pathway of apoptosis, was performed [34]. Finally, to determine whether ferroptosis occurred in ECOs, ACSL4 expression was analyzed by qRT-PCR.

RNA was isolated from the organoids using the RNeasy Micro Kit (ref. #74034, Quiagen, NL). Complementary DNA was generated using the Quanti Nova SYBR Green Kit (ref. #208056, Quiagen, NL), and gene expression was determined by quantitative real-time PCR (qRT-PCR) using the LightCycler 480 (ref. # 05015278001, Roche Diagnostics, CH). Primers used were for ACSL4 (GeneGlobe ID: QT00040992), HIF-1α (GeneGlobe ID: QT00083664), VEGF A (GeneGlobe ID: QT01682072), SLC2A1 (GeneGlobe ID: QT00068957), cyclin D1 (GeneGlobe ID: QT00495285), LGR5 (GeneGlobe ID: QT00027720), SOX-9 (GeneGlobe ID: QT00223055), EpCAM (GeneGlobe ID: QT00000371), albumin (GeneGlobe ID: QT00063693), BAX (GeneGlobe ID: QT00031192), Bcl-2 (GeneGlobe ID: QT00025011), Caspase 3 (GeneGlobe ID: QT00023947)and CK-19 (GeneGlobe ID: QT00081137) (ref. # 249900, Quiagen, NL).

#### Immunohistochemistry, Immunofluorescence and mIFISH

ECOs and bile duct specimens were formalin-fixed and paraffin-embedded (FFPE). FFPE blocks were cut into 4 µM sections and standard protocols were used [35]. Specifications are listed in Supplementary Table S1. Immunohistochemistry (IHC) and immunofluorescence (IF) staining’s were performed with a standard protocol [1, 33]. Multiplex immunofluorescence and *in-situ* hybridization (mIFISH) was performed as described before [35]. mIFISH allows both the detection of cytokines through *in-situ* hybridization and parallel phenotyping of cells through immunofluorescence staining, thus showing the cellular origin of cytokines within FFPE specimens [35]. Negative controls are provided in Supplementary Figures S1–S3.

#### *In-Situ* Hybridization

Chromogenic *in-situ* hybridization (cISH) of FFPE embedded ECOs was performed with the RNAscope HD-RED Assay (ref. #322350, ACD, Bio-Techne, USA), according to the manufacturer’s instructions; only the incubation time of the Amplification 5 was modified by extending to 1 h. As a positive control to assess the presence and abundance of RNA in the samples, we used *homo sapiens* ubiquitin C: UBC (ref. #310041, ACD, Bio-Techne, United States). Negative controls are provided in Supplementary Figures S4, S5.

#### Automated Histology Image Analysis

Whole slide scans were obtained using PANNORAMIC 1000 (3DHISTECH, HU). For the automated whole slide quantitative image analysis, we wrote an analysis algorithm using the open-source software QuPath v.0.4.3 [34]. First, a simple tissue detection algorithm was used to distinguish the sample from background. Second, a pixel classifier based on an artificial neural network was trained to further delimit regions containing biliary epithelium, as well as peri-biliary glands. After this step, all slides were manually checked, and false positive detections manually deleted to ensure that all remaining ROIs contained only biliary epithelial cells. Third, a cell detection algorithm was employed to distinguish single cells within the previously defined regions of interest based on nuclei detection and an estimation of cell area (Supplementary Figure S6).

For samples employing *in-situ* hybridization staining, quantitative analysis was performed using a subcellular detection algorithm which allows for a distinction between single detections of mRNA products and detection of clusters. The count of single mRNA products per cluster was estimated based on the size and cluster intensity.

Samples treated by immunohistochemical stains were analyzed using a positive cell detection algorithm, resulting in an H-Score for every region of interest as well as for the entire sample. All steps were manually checked for plausibility and validity by two independent researchers (PK and HJ).

This approach is supported by existing literature showing that qPCR and automated image analysis for *in-situ* hybridization had good correlation [36, 37]. Furthermore, automated image analysis for immunohistochemical stains using H-scores correlate with qPCR [38–40].

#### Statistical Methods

Statistical analysis was performed using Prism 10 software (GraphPad, Dotmatics, USA). For all data sets, normal distribution was tested using the Shapiro-Wilk test. For interval-scaled variables, one-way ANOVA was used followed by a corrected Dunn´s test to correct for alpha error when comparing multiple groups.

## Results

### IRI in ECOs Mimics IRI Found in Common Bile Duct Specimens Obtained During LT

ECOs expressed EpCAM, CK19, ZO-1 and SOX-9, but did not stain with monoclonal mouse anti-human hepatocyte antibody (AHH) (Figure 1B, 1top row). Human bile duct biopsies also expressed EpCAM, CK19, ZO-1 and SOX-9 while not staining for AHH (Figure 1B, bottom row). Conversely, human liver samples did not stain for biliary markers while staining positively for AHH (Figure 1B, middle row). GGT1 is expressed in the liver sample while no GGT1 could be detected in either ECOs or extrahepatic bile duct biopsies (Figure 1B, third column). Protein expression for key markers was confirmed by qRT-PCR (EpCAM, CK19, LGR5, SOX-9), whereas albumin as marker for hepatocytes was not expressed in ECOs (Figure 1C). Conversely, the hepatocellular cell line HEPA RG did not express these cholangiocyte markers in qRT-PCR analysis, while albumin was highly expressed.

With cholangiocyte characteristics confirmed, ECOs were cultured under hypoxic conditions for 48 h to simulate low oxygen conditions during organ ischemia; they were subsequently reoxygenated for 24 h to simulate organ reperfusion. Under normoxic conditions, ECOs showed a regular organoid architecture and a regular cylindric epithelia cell lining (Figure 1D, left top and middle panel). The cylindric bile duct epithelium in ECOs is comparable to undamaged extrahepatic bile duct epithelium (Figure 1D, left bottom panel). Organoid disruption occurred after 48 h of hypoxia (Figure 1D, top right panel), and epithelial damage in ECOs with flattened epithelial cells (Figure 1D, right middle panel) was like the epithelial damage observed in the extrahepatic bile during LT (Figure 1D, right bottom panel). Therefore, 48 h of hypoxia was used in the subsequent *in-vitro* experiments.

### Extrahepatic Bile Duct Specimens and ECOs Show Similar Responses to Ischemic Stress

To test whether ECOs respond to hypoxia and reoxygenation in a similar way as actual real-life bile ducts during LT, we assessed the response of extrahepatic bile ducts (non-diseased bile duct, cold storage, reperfusion) to hypoxic conditions by investigating the molecular response to ischemia and reperfusion. HIF-1α protein expression was assessed by IHC and at a transcription level by qRT-PCR.

**FIGURE 2:**
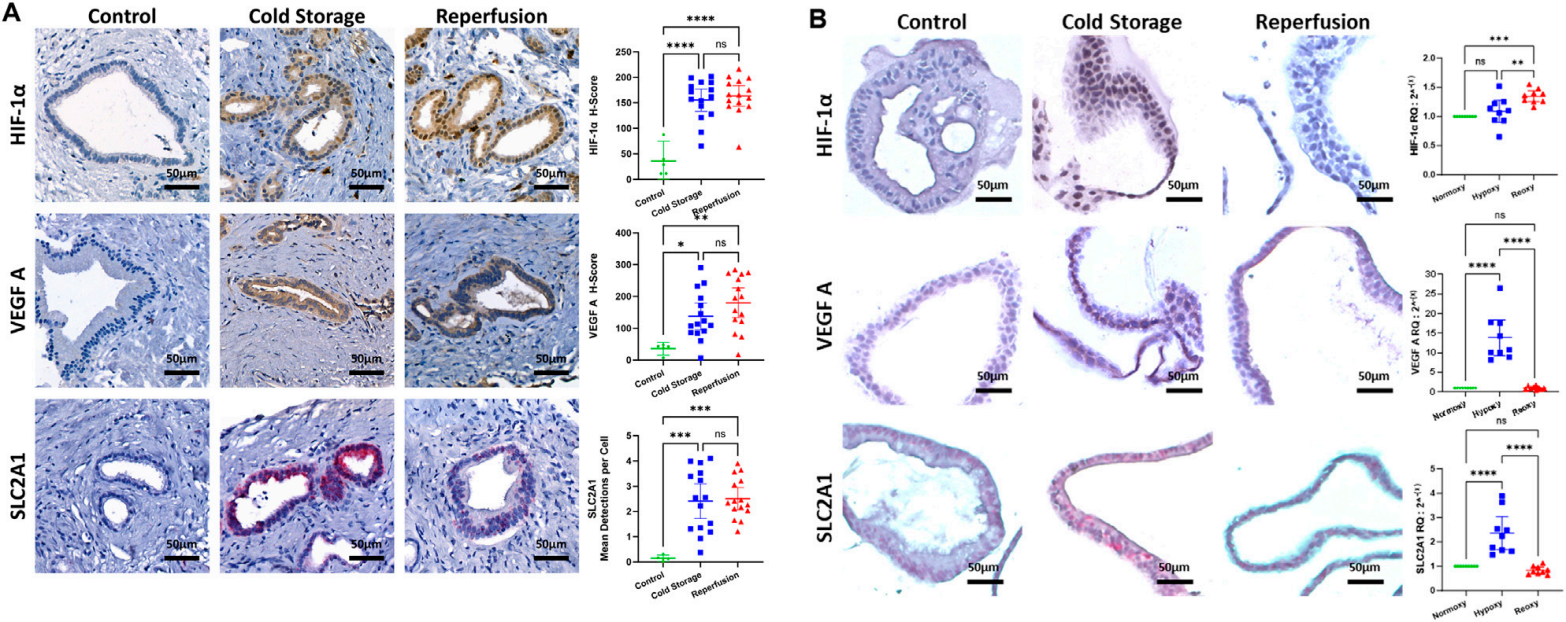
Ischemic stress in the extrahepatic bile duct during liver transplantation and ECOs. **(A)** Representative stain for HIF-1α (IHC, top row), VEGF A (IHC, middle row) and SLC2A1 (cISH, bottom row) in the extrahepatic bile duct. Whole slide automated image analysis showed HIF-1α, VEGF A and SLC2A1 expression increased in cold storage and reperfusion biopsies, compared to controls. **(B)** Representative stains for HIF-1α (IHC, top row), VEGF A (IHC, middle row) and SLC2A1 (cISH, bottom row) in ECOs. qRT-PCR showed an increase in HIF-1α mRNA expression between normoxy and reoxygenation, and between hypoxia and reoxygenation; no differences between normoxy and hypoxia were seen. qRT-PCR showed an increase in VEGF A and SLC2A1 expression between normoxy and hypoxia, and a decrease between hypoxia and reoxygenation. (ns: non-significant, * p < 0.05, ** p < 0.01, *** p < 0.001, **** p < 0.0001).

In the extrahepatic bile duct during LT, we observed an increase in HIF-1α on a protein level in the cold storage and reperfusion biopsies, compared to the control (Figures 2A, top row). Whole slide automated image analysis confirmed that HIF-1α expression in these cold-stored and reperfused samples increased compared to controls. However, we did not observe an additional increase in HIF-1α protein after reperfusion when compared to the cold storage condition (Figures 2A, top row). In ECOs, we did not observe an increase in HIF-1α protein expression during the hypoxia phase but did see an increase after reoxygenation; qRT-PCR confirmed this result on mRNA level (Figures 2B, top row).

The extrahepatic bile duct biopsies obtained during cold storage and after reperfusion displayed an increase in VEGF A protein assessed through IHC, and SLC2A1 mRNA assessed through cISH when compared to control biopsies (Figures 2A, VEGF A middle/ SLC2A1 bottom row). There was no difference in VEGF A and SLC2A1 expression between cold storage and reperfusion conditions. Levels of VEGF A (Figure 2B, middle row) and SLC2A1 (Figure 2B, bottom row) mRNA expression in ECOs both increased under hypoxic condition, indicating that adequate hypoxia in ECOs was achieved. After ECOs were reoxygenated for 24 h, mRNA levels of VEGF A and SLC2A1 both returned to baseline levels, illustrating the capacity of ECOs to recover from hypoxic damage.

### Cell Death in LT Setting

**FIGURE 3:**
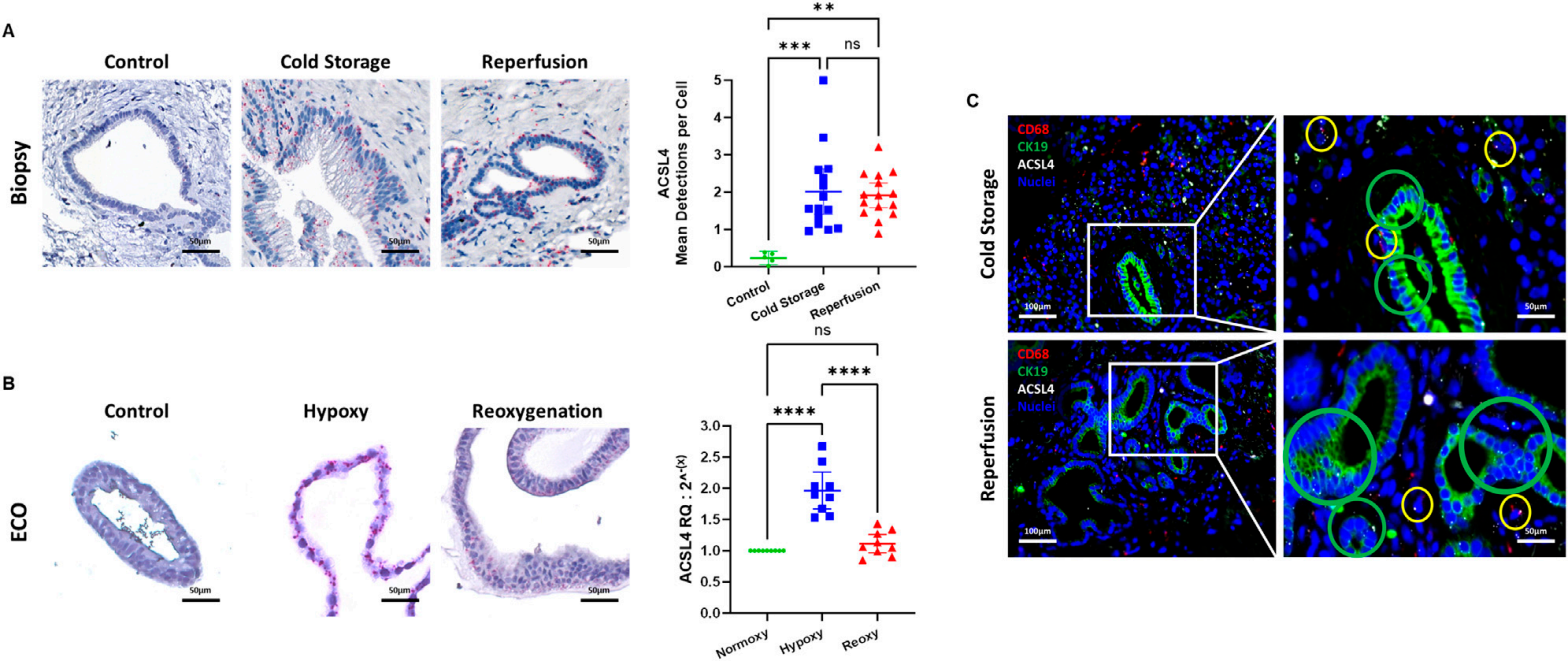
Ferroptosis in liver transplant setting. **(A)** ACSL4 mRNA expression in the extrahepatic bile duct quantified by cISH. At baseline (non-diseased gall bladder), no ACSL4 expression was detected. During static cold storage conditions and after reperfusion, high expression of ACSL4 in the PBG of the extrahepatic bile duct was observed. Fully quantitative computer assisted image analysis of the cISH displays an increase of ACSL4 between the control and both cold storage biopsies and the reperfusion biopsies. **(B)** ACSL4 mRNA expression in the extrahepatic bile duct quantified by cISH and qRT-PCR. Control ECOs showed no ACSL4 expression in the cISH, whereas ACSL4 expression increased during hypoxic conditions. After reoxygenation, no ACSL4 expression was observed. qRT-PCR showed a significant increase in ACSL4 expression during hypoxia, whereas a significant decrease was observed after reoxygenation. **(C)** Multiplex immunofluorescence and *in-situ* hybridization (mIFISH) of biopsies obtained during cold storage, as well as after reperfusion. ACSL4 is expressed by CD68^+^ macrophages (yellow circle) and by CK19^+^ cholangiocytes (green circle). (ns: non-significant, ** p < 0.01, *** p < 0.001, **** p < 0.0001).

We analyzed common bile duct biopsies using cISH and observed increased ACSL4 expression during cold storage and after reperfusion in the peribiliary glands, compared to healthy controls (Figure 3A). In ECOs, we observed an increase in ACSL4 in the hypoxia group that dopped back down to baseline levels after ECOs were re-oxygenated for 24 h (Figure 3B). We suggest this effect is due to the capacity of ECO cells to recover from hypoxia when being reintroduced to normoxic conditions. We further performed a multiplex (mIFISH) staining to identify the cellular source of ACSL4 in the extrahepatic bile duct. Interestingly, both cholangiocytes (CK19^+^) and macrophages (CD68^+^) expressed ACSL4 during cold organ storage and after liver reperfusion (Figure 3C). Together, these data suggest that ferroptosis pathways are induced during the transplant procedure and therefore can contribute to IRI in the extrahepatic bile duct, and that ECOs mimic upregulation of the ferroptosis trigger in extrahepatic bile ducts.

**FIGURE 4:**
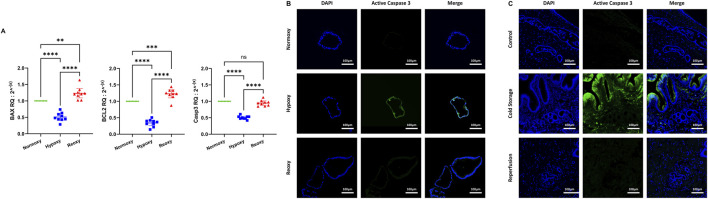
Apoptosis in liver transplant setting. **(A)** mRNA expression for BAX, Bcl-2 and Caspase 3 all decreased significantly during hypoxia. While there was no significant increase in Caspase 3 mRNA levels after reoxygenation when compared to normoxy conditions, mRNA levels of Bcl-2 and BAX at reoxygenation were significantly elevated compared to normoxy conditions. (ns: non-significant, ** p < 0.01, *** p < 0.001, **** p < 0.0001). **(B)** Apoptotic activity in ECOs was observed in an IF stain for active caspase 3. While there was no signal detected for active caspase 3 in both normoxic and reoxygenated ECOs, a signal for active caspase 3 was detected in hypoxic ECOs. **(C)** Immunofluorescence for active caspase 3 was performed in bile duct biopsies. There is no active caspase 3 present in control biopsies. In biopsies obtained during cold storage, active caspase 3 can be observed, especially in biliary epithelial cells. One hour after reperfusion, very little active caspase 3 can be detected.

Regarding apoptosis, mRNA levels of pro-apoptotic BAX, anti-apoptotic Bcl-2 and caspase 3 all significantly dropped after 48 h of hypoxia with caspase 3 mRNA levels returning to baseline after reoxygenation while levels of BAX and Bcl-2 increased significantly above baseline after reoxygenation (Figure 4A). We then performed IF staining for active caspase 3 in both ECOs (Figure 4B) and biopsies (Figure 4C). Hypoxia respectively ischemia led to activation of caspase 3 in both ECOs and biopsies with levels dropping back down after reoxygenation/reperfusion.

### Proliferative Activity in ECOs

**FIGURE 5:**
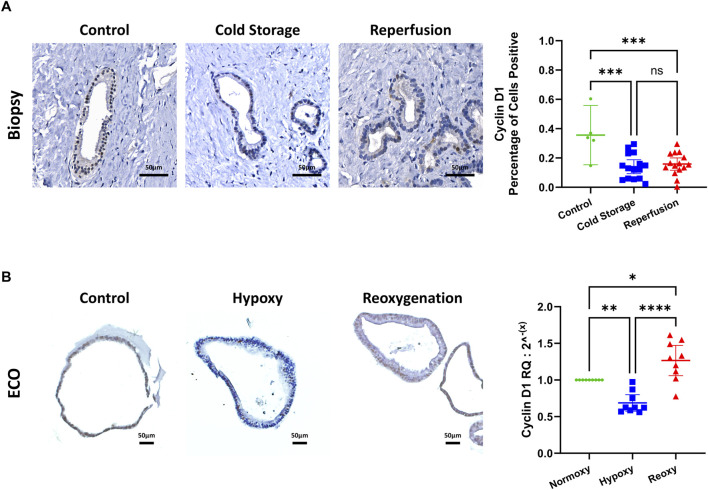
Proliferative activity assessed by cyclin D1. **(A)** Representative cyclin D1 IHC stain of biopsies of the common extrahepatic bile duct, with automated computer assisted analysis. IRI leads to decreased cyclin D1 expression in the extrahepatic bile duct. **(B)** Representative cyclin D1 IHC staining of ECOs (left) and mRNA expression measured by qRT-PCR (right). Reduced cyclin D1 expression under hypoxic conditions, with an increase after reoxygenation. (ns: non-significant, * p < 0.05, ** p < 0.01, *** p < 0.001, **** p < 0.0001).

To assess proliferative activity, we used cyclin D1. We determined cyclin D1 levels through IHC and fully automated computer assisted image analysis in bile duct biopsies and through qRT-PCR in ECOs. In bile duct biopsies, cyclin D1 positive cells are decreased after cold storage and after reperfusion when compared to control biopsies (Figure 5A). In ECOs we observed a decrease in cyclin D1 during hypoxia when compared to baseline, but after reoxygenation for 24 h, cyclin D1 concentrations increased compared to both baseline and hypoxia (Figure 5B).

### Disruption of Epithelial Cohesion and Architecture

**FIGURE 6:**
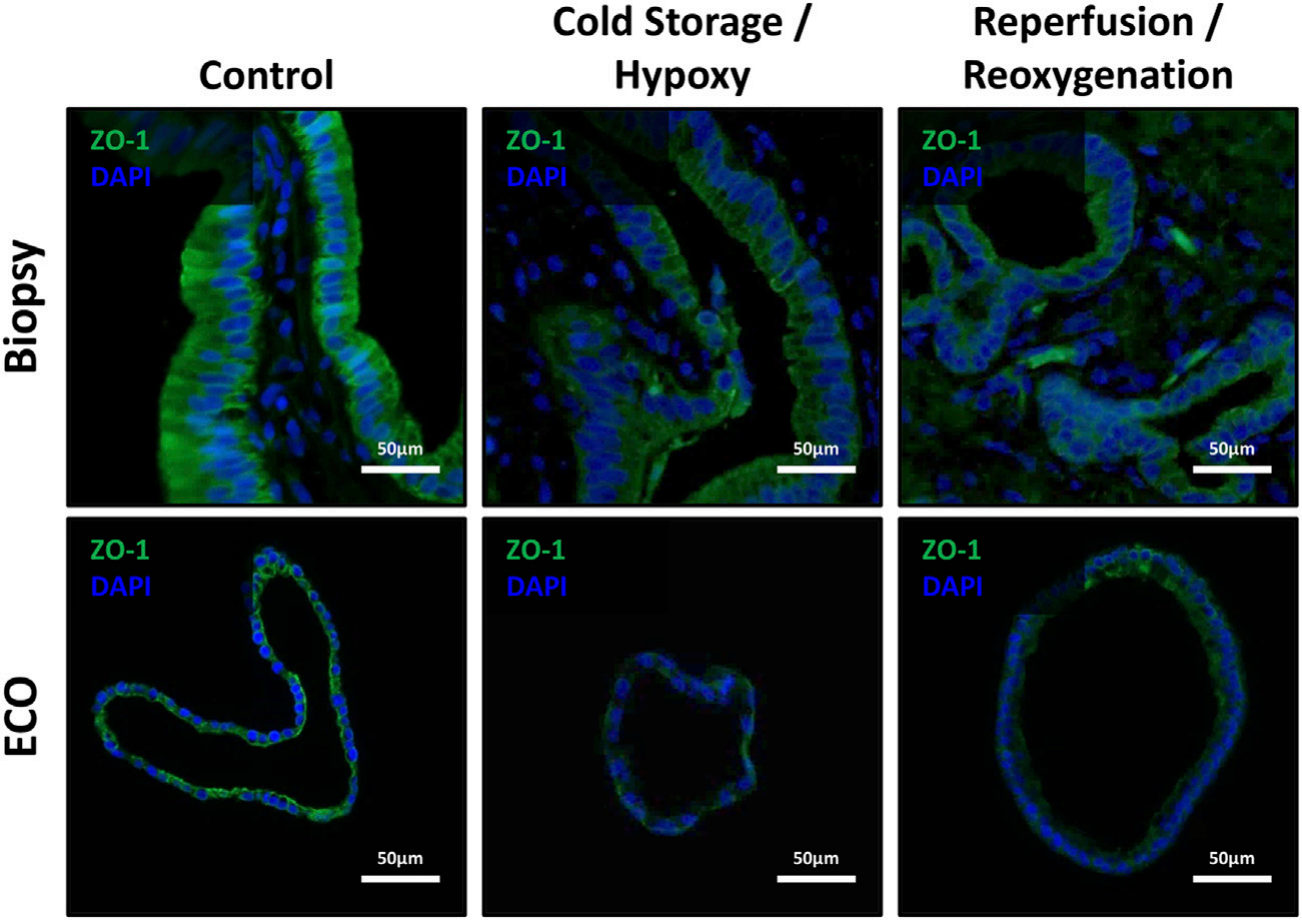
ZO-1 expression showed disruption of epithelial cohesion and architecture. *Top row:* Representative IF stains of ZO-1 in bile duct specimens revealed the loss of ZO-1 from non-ischemic conditions over cold storage to the reperfusion with an observable loss of epithelial cohesion and epithelial architecture. *Bottom row*: ECOs show a similar behavior, except for the reoxygenation condition where some recovery of ZO-1 and epithelial organization and cohesion is present.

Since IRI leads to epithelial cell architecture disruption in common bile ducts after transplantation [1], we investigated expression of Zonula occludens-1 protein in ECOs and bile duct biopsies. Cohesion of bile duct epithelial cells was disrupted in both biopsies and ECOs, along with a decrease and uneven expression of ZO-1 in these cells during cold storage/hypoxia and reperfusion/reoxygenation (Figure 6), respectively. Notably, reoxygenated ECOs showed signs of epithelial reorganization and ZO-1 regeneration, suggesting the beginning of a recovery phase in stressed biliary epithelium. Bile duct biopsies also display levels of epithelial disruption up to complete loss of biliary epithelium in both cold storage and the reperfusion condition. No reorganization of epithelium can be observed in biopsies, which is likely due to the short time span after reperfusion until reperfusion biopsies were obtained.

## Discussion

The aim of the present study was to introduce a novel organoid model using extrahepatic cholangiocyte organoids to mimic the complex pathophysiological effects to the biliary system during LT. To assess the usefulness of our model, we show that: a) the cultivated ECOs are of biliary phenotype, b) adequate hypoxia has been achieved in ECOs and that similar hypoxic conditions are present in human extrahepatic bile ducts during LT, c) ferroptosis is triggered in ECOs and human bile ducts, and d) cell proliferation is present in bile ducts and ECOs under IRI conditions, indicating regenerative activity.

Confirmation of biliary phenotype was the first aim of our study. Markers for ductal and biliary phenotype such as EpCAM, CK19 and SOX-9 were present in ECOs and healthy biliary biopsies in accordance with existing literature [11, 12, 14]. Furthermore, ECOs expressed ZO-1, which, according to existing literature, is key in barrier functionality of biliary epithelium [41, 42]. A point of note is that ECOs also expressed LGR5, which is part of the Wnt/β-catenin pathway [43] and a proliferative marker commonly found in adult stem cells of various origin [14, 44–46]. In human bile ducts, stem cells are located in the peribiliary glands [47, 48]. GGT1 could be detected in neither ECOs nor extrahepatic bile duct samples, especially the peribiliary glands, while being present in the healthy liver tissue. This contradicts the findings of Sampaziotis et al. who found GGT expression and activity in their biliary organoid cultures [11, 12]. However, Rimland, Tilson et al. noted a downregulation of GGT1 when the Wnt/β-catenin pathway is active in biliary organoids [14]. Furthermore, they noted that canonical activation of the Wnt/β-catenin signal pathway led to LGR5 expression in extrahepatic bile duct organoids giving them adult stem cell properties [14]. Since LGR5 expression was found in ECOs, that might explain the absence of adequate GGT1expression. While ECOs behaved like biliary epithelial cells in our experiments, they also possess the regenerative proliferative potential of cholangiocytes found in the peribiliary glands of the extrahepatic common bile duct, previously described by DeJong, Matton et al. [48]. Our results suggest that ECOs provide a suitable *in-vitro* system to study IRI, since they consist of cholangiocytes and have progenitor cell properties that can be subjected to hypoxia and subsequent reoxygenation.

Measuring hypoxia in ECOs proved challenging. One of the most sensitive known intrinsic cellular responses is the expression of HIF-1α [16, 49, 50]. The expression pattern of HIF-1α observed in qRT-PCR analysis of ECOs is consistent with existing research, since HIF-1α is transcribed into mRNA at constant levels [51, 52] and HIF-1α concentrations are regulated mainly through oxygen-dependent degradation of HIF-1α protein (via von Hippel-Lindau protein and prolyl hydroxylases) [16, 50]; there is no direct upregulation of HIF-1α transcription prior to HIF-1α protein increases under hypoxic conditions [51, 52]. However, the increase in HIF-1α transcription after reoxygenation is also in accordance with existing literature where epigenetic modifications trigger intermittent hypoxia [51].

To further show that adequate hypoxia was achieved, we focused on downstream indicators of a HIF-1α protein accumulation as a result of reduced degradation. Indeed, neoangiogenesis is driven in hypoxic tissues by a number of key growth factors, including VEGF A, which is important in tissues undergoing injury and regeneration [16, 53, 54]. Furthermore, SLC2A1 is a significant transporter under hypoxic conditions, allowing hypoxic cells relying on anaerobic glycolysis to transport glucose and lactate [16, 29, 30].

Hypoxia and regeneration experiments were helpful to further characterize and contrast ECOs to bile duct biopsy material collected during LT. We found that after hypoxia, ECO-epithelium showed similar morphological changes to that observed in bile ducts during LT [1, 55, 56]. The IRI in ECOs after hypoxia and reperfusion stress further led to the disruption of Tight-Junction-Protein 1 in epithelial cells, as previously described in bile duct specimens after LT [1]. When compared to the human bile duct specimens obtained during LT, biomarkers for hypoxia in biopsies after cold storage behaved similarly to ECOs under hypoxic conditions. However, after reperfusion, biomarkers in bile duct biopsies obtained after reperfusion did not drop back down as expected and seen in ECOs after reoxygenation. Thus, we speculate, that the observed differences could be attributed to the relatively short period of reperfusion (<1 h) before the biopsies were taken, which is prior to the biliary anastomosis. A longer timespan can’t be advocate due to patient safety.

While ferroptosis is thought to contribute to IRI in various organs [18, 20–22, 57–59], the role of ferroptosis in biliary system context is unknown. In the present study we show ferroptotic activity in cholangiocytes of the extrahepatic bile duct during LT. ECOs mimic the upregulation of the major ferroptosis trigger ACSL4 in extrahepatic bile ducts during the hypoxic phase. In contrast to the biopsies, ECOs returned to baseline after 24 h of reoxygenation. We speculate, that the observed differences could be attributed to the relatively short period of reperfusion (<1 h) before the biopsies were taken, which is prior to the biliary anastomosis, compared to 24 h of reoxygenation of ECOs. Together, these data suggest that ferroptosis pathways are induced during the transplant procedure and therefore can contribute to IRI in the extrahepatic bile duct, and that ECOs mimic upregulation of the ferroptosis trigger in extrahepatic bile ducts.

Additionally, we investigated apoptosis in ECOs. mRNA expression of factors belonging to the intrinsic pathway of apoptosis (BAX and Bcl-2were downregulated in ECOs under hypoxia and upregulated upon reoxygenation. This finding is consistent with existing literature [25]. Expression of caspase 3, a member of the common pathway of both intrinsic and extrinsic apoptotic signaling [34] was downregulated after 48 h of hypoxia but returned to baseline upon reoxygenation. To assess whether apoptosis was active in cells, we assessed active caspase 3 in both ECOs and biopsies finding a similar pattern of apoptotic activity in both. This further supports the hypothesis that ECOs are indeed a suitable model for studying IRI *in vitro*.

Lastly, our aim was to show, that proliferative activity in ECOs was present indicating the initiation of regenerative processes that matches that found in peribiliary glands *in-vivo*. Indeed we found that after a decrease in proliferative activity during hypoxia, which can be reconciled with existing literature both *in-vitro* and *in-vivo* [25, 48]. After reoxygenating the organoids, proliferative activity exceeded baseline. This also is congruent with findings from previous *in-vivo* studies [48]. Furthermore, that study provides a reason as to the lack of increase in Cyclin D1 in bile duct biopsies which can again be attributed to the short interval between start of reperfusion and procurement of the reperfusion biopsy specimen with a significant increase in proliferation taking up to 48 hrs. In-vivo [48].

While an *in-vitro* organoid model has been established previously using intrahepatic cholangiocytes [25], no such model exists to date that has been compared directly to bile duct specimens obtained during LT at hypoxia (cold storage condition) and after reoxygenation (reperfusion). Moreover, research on cholangiocyte organoids has shown that intrahepatic and extrahepatic cholangiocytes are morphologically different [14], warranting the development of this model using extrahepatic cholangiocytes. This model opens the way to investigate many aspects of bile duct pathophysiology, including studying impact of critical mediators of inflammatory and subsequent regenerative responses that are so critical for repairing damage caused by IRI in LT patients.

## Figures and Tables

**Table:** 

**ACSL4**	Acyl-CoA Long Chain Family Member 4
**AHH**	Anti-Human Hepatocyte
**ANOVA**	Analysis of variance
**Bcl-2**	B-cell CLL/lymphoma 2
**BAX**	Bcl-2-associated X protein
**CISH**	Chromogenic *in-situ* hybridization
**CIT**	Cold ischemia time
**CK19**	Cytokeratin-19
**CO**_**2**_	Carbon Dioxide
**DNA**	Deoxyribonucleic acid
**ECOs**	Extrahepatic cholangiocyte organoids
**EpCAM**	Epithelial cell adhesion molecule
**FFPE**	Formalin Fixed and Paraffin Embedded
**Fig**	Figure
**GGT1**	Gamma-glutamyltransferase 1
**H/R**	Hypoxia/Reper
**HIF-1α**	Hypoxia-inducible factor 1-alpha
**I/R**	Ischemia/Reperfusion
**IF**	Immunofluorescence
**IHC**	Immunohistochemistry
**IRI**	Ischemia/reperfusion injury
**LGR5**	Leucine-rich repeat-containing G-protein coupled receptor 5
**LT**	LT
**mIFISH**	Multiplex immunofluorescence and *in-situ* hybridization
**Min**	Minutes
**qRT-PCR**	Quantitative real-time PCR
**RNA**	Ribonucleic acid
**SOX-9**	Transcription factor SOX-9
**Suppl**	Supplementary
**VEGF A**	Vascular endothelial growth factor A
**ZO-1**	Zonula occludens-1

## Data Availability

The raw data supporting the conclusions of this article will be made available by the authors, without undue reservation.
