# Supplementary figures and images for: Extrahepatic Bile Duct Organoids as a Model to Study Ischemia/Reperfusion Injury During Liver Transplantation
Source: Transpl Int. 2024 Sep 11;37:13212. doi: 10.3389/ti.2024.13212 (PMC11422091; doi:10.3389/ti.2024.13212)

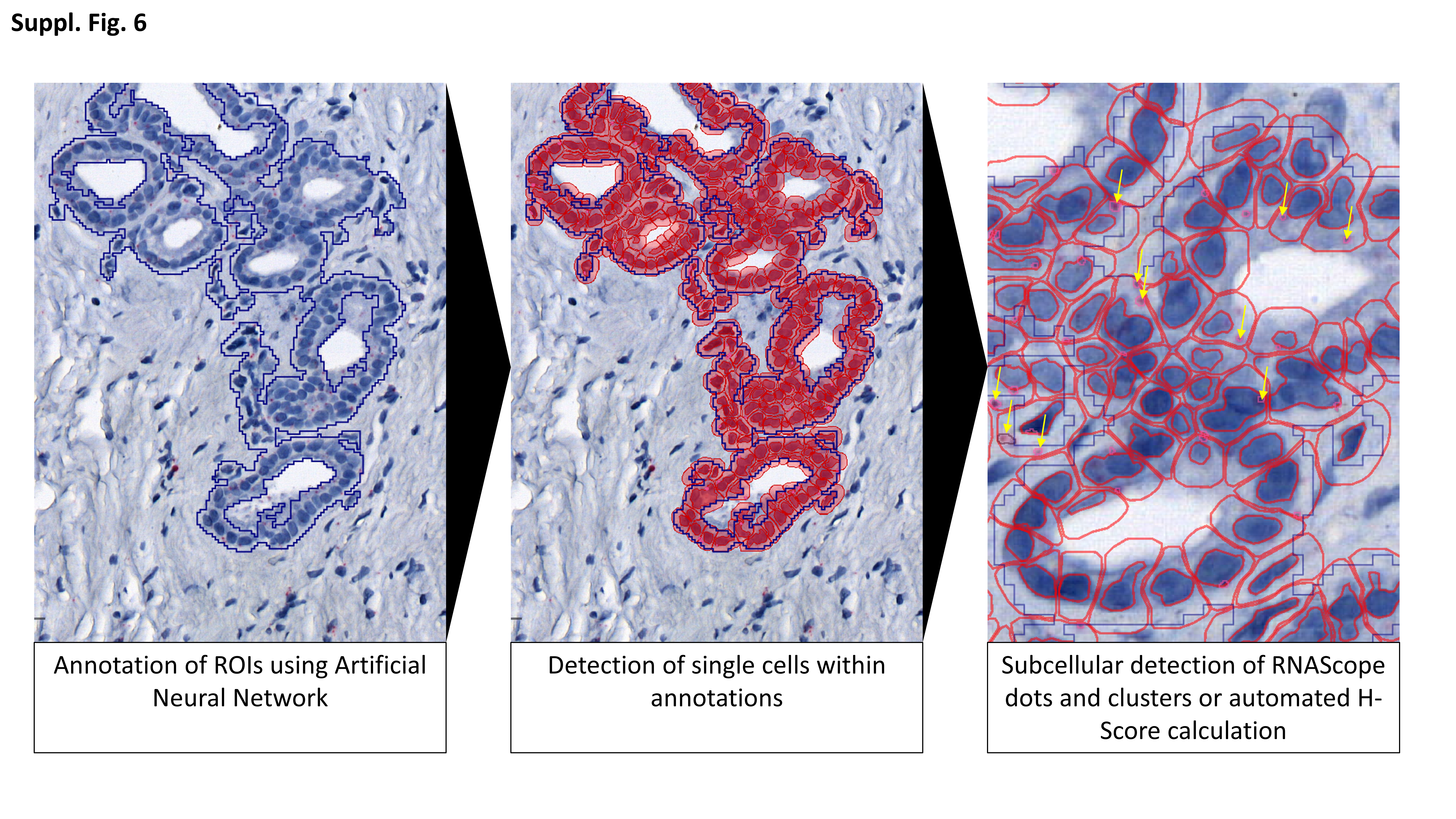

Supplement: Supplementary file 1 [file Image6.TIF]

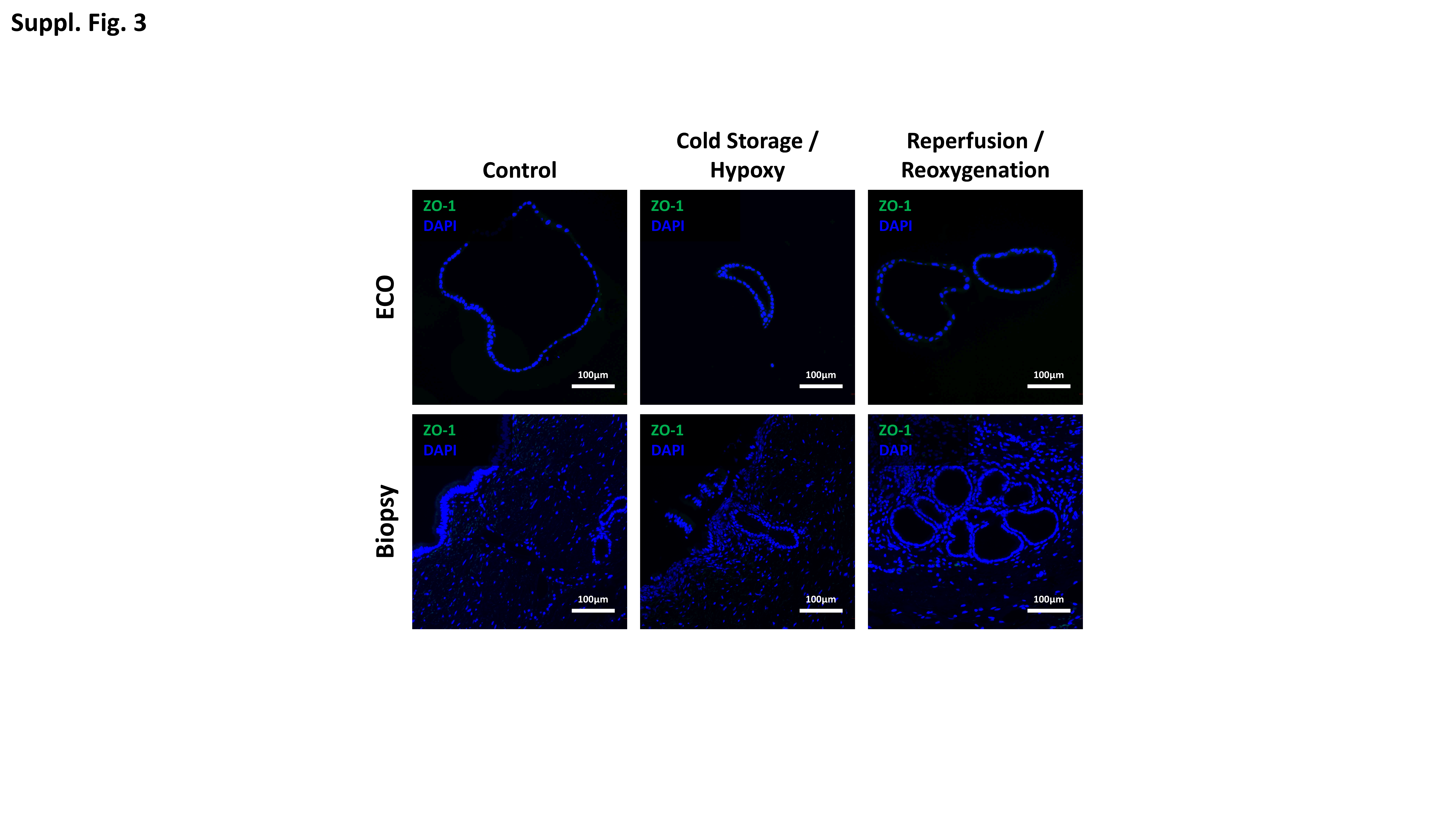

Supplement: Supplementary file 3 [file Image3.TIF]

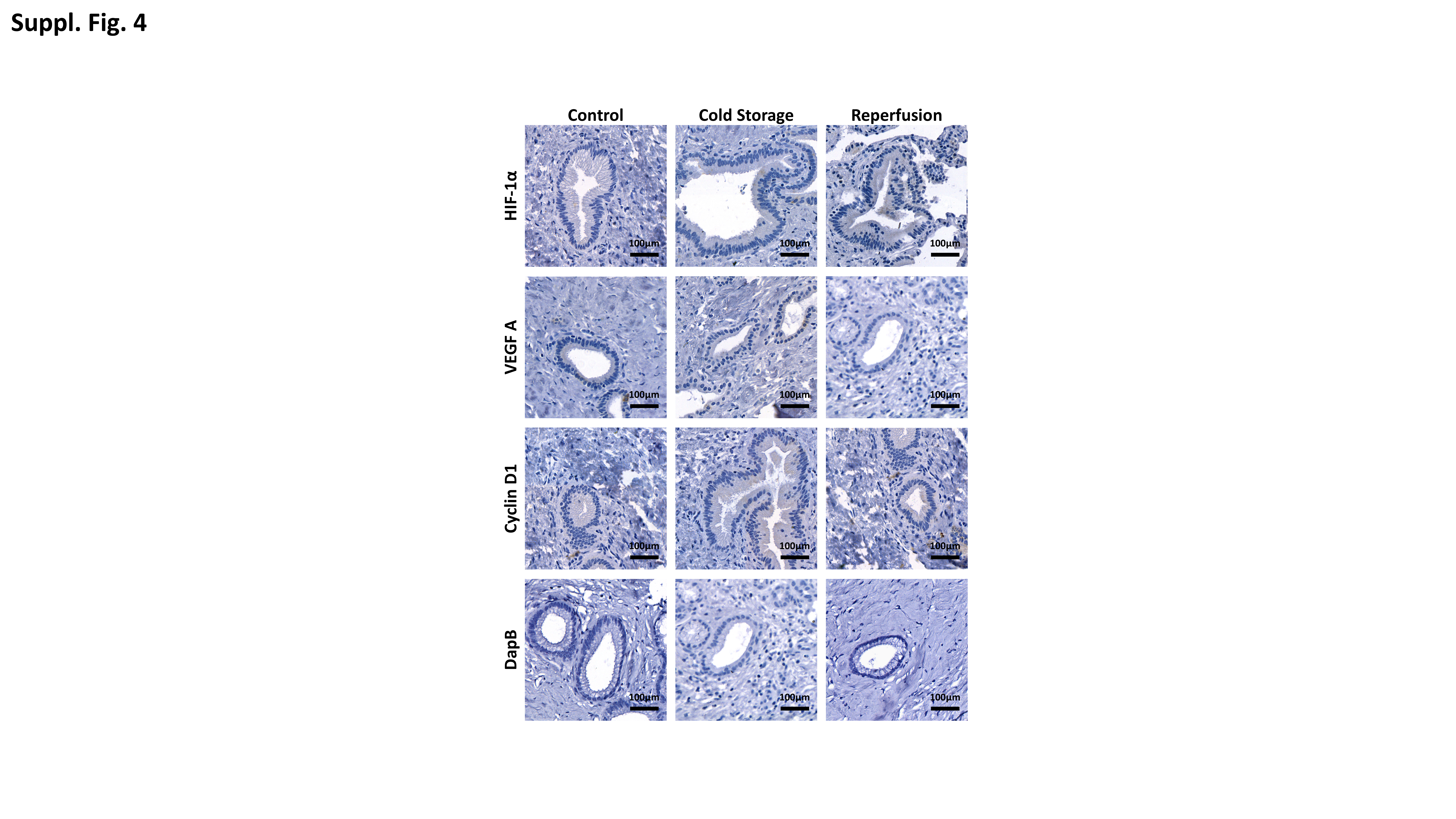

Supplement: Supplementary file 4 [file Image4.TIF]

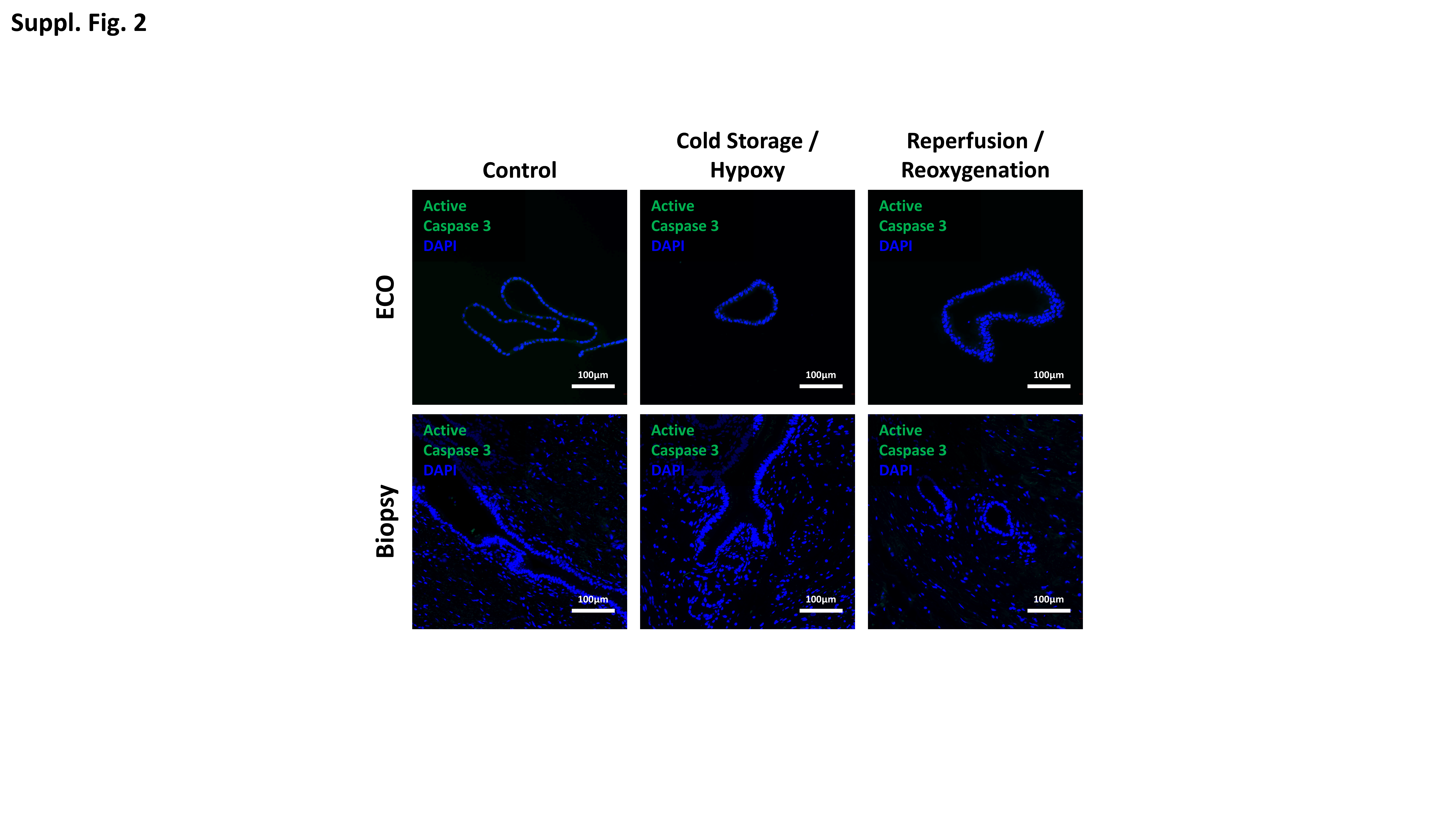

Supplement: Supplementary file 5 [file Image2.TIF]

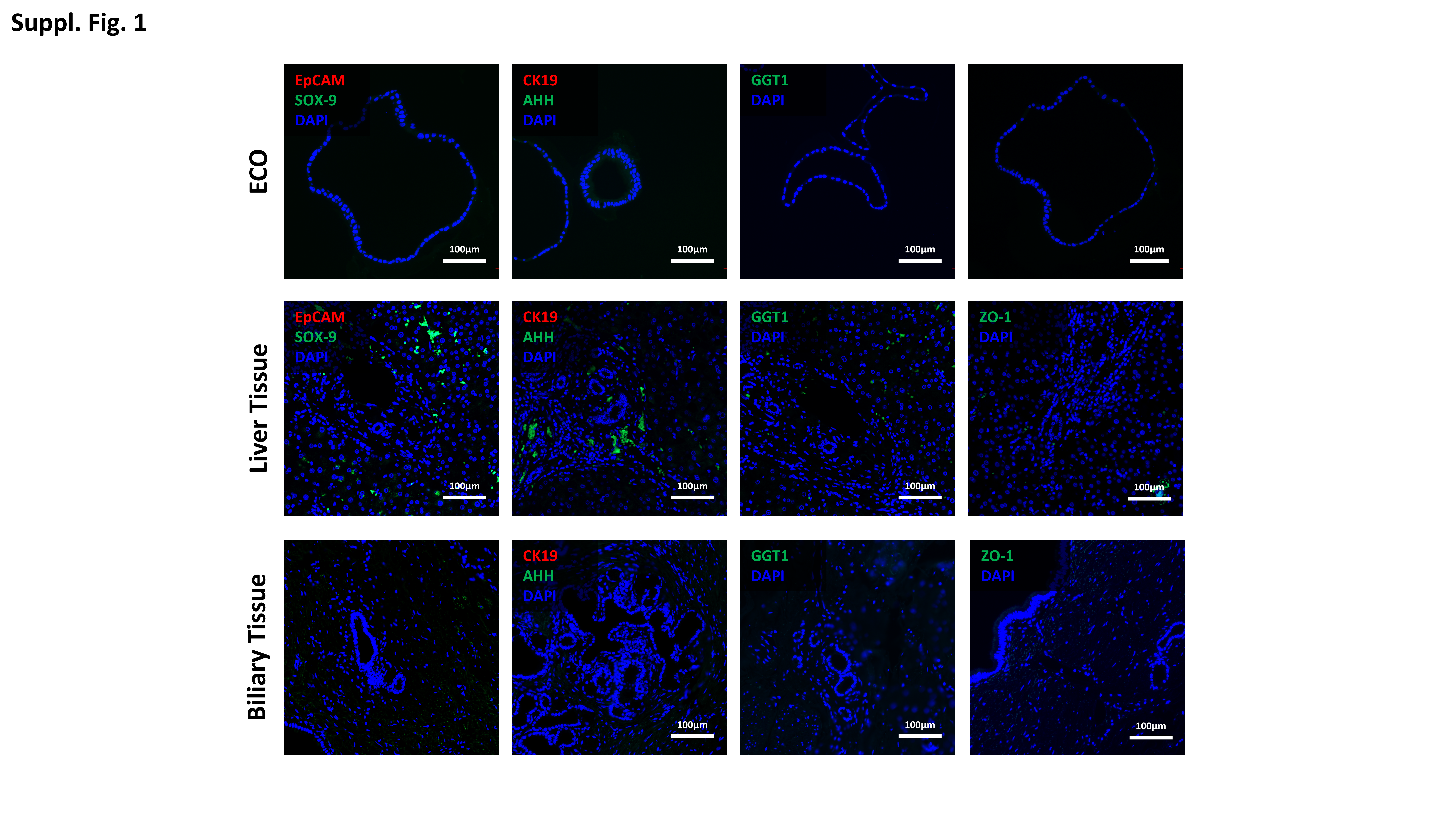

Supplement: Supplementary file 6 [file Image1.TIF]

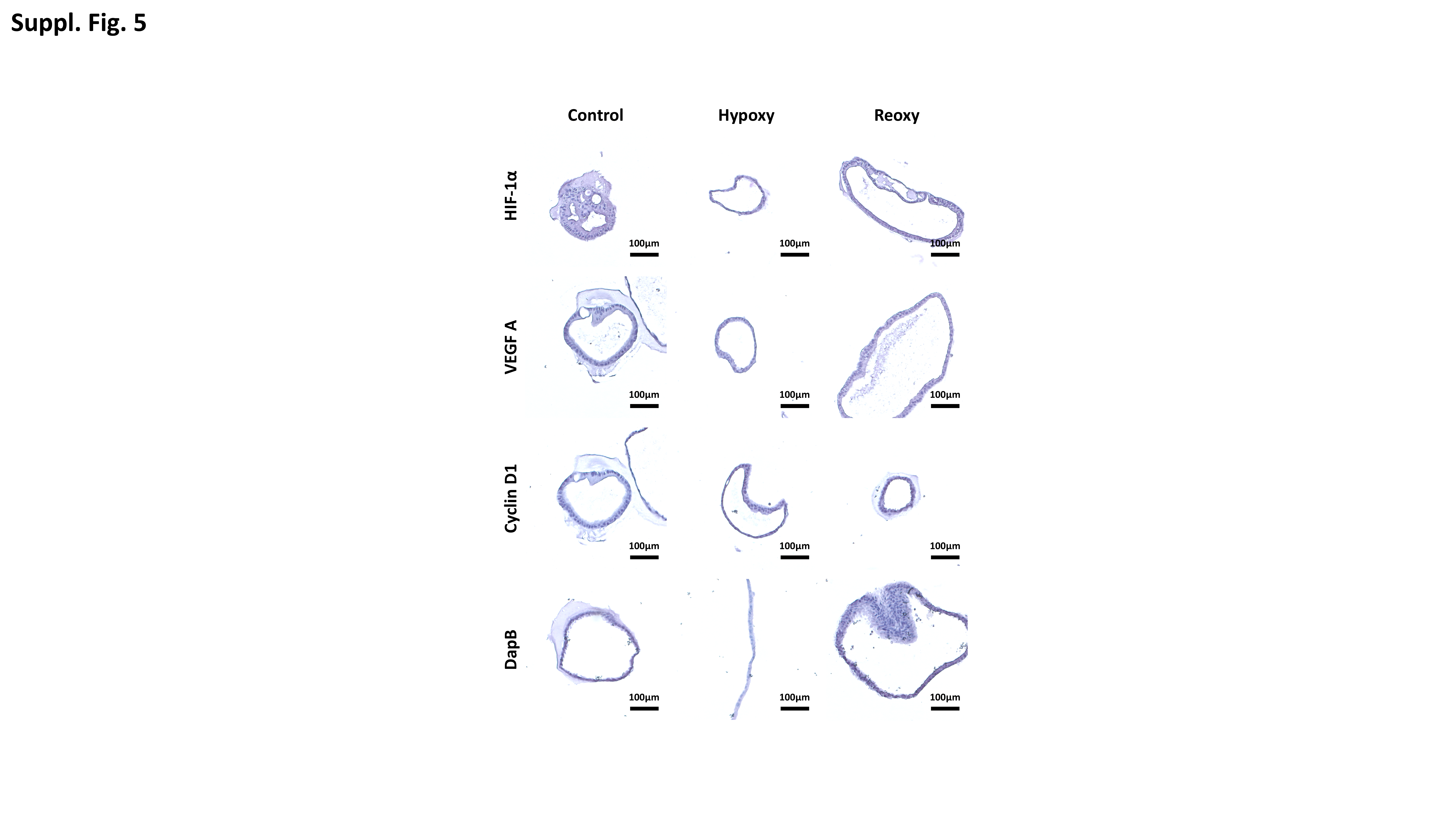

Supplement: Supplementary file 8 [file Image5.TIF]
