# Supplementary material for: Extrahepatic Bile Duct Organoids as a Model to Study Ischemia/Reperfusion Injury During Liver Transplantation
Source: Transpl Int. 2024 Sep 11;37:13212. doi: 10.3389/ti.2024.13212 (PMC11422091; doi:10.3389/ti.2024.13212)
Supplement: Supplementary file 2 [file Table1.docx]

| **Suppl. Table 1: Donor Descriptives** | | | |
| --- | --- | --- | --- |
|  | **Mean ± SD / Total Number (Percentage)** |  | **Mean ± SD / Total Number (Percentage)** |
| **Donor Age (years)** | 53.68 ± 18.42 | **Donor Type**  **DBD**  **DCD** | 15 (100.00%)  0 (0.00%) |
| **Donor Sex**  **Male**  **Female** | 5 (33.33%)  10 (66.66%) | **Time of Cardiac Arrest/Hypotension (min)** | 1.53 ± 4.95 |
| **Donor Height (cm)** | 164.47 ± 19.94 | **Organ Quality**  **Good**  **Acceptable**  **Marginal** | 13  2  0 |
| **Donor Weight (kg)** | 75.73 ± 20.78 | **Perfusion Solution HTK UW** | 13 (86.67%)  2 (13.33%) |
| **Donor BMI (kg/m^2^)** | 27.02 ± 4.79 | **Warm Ischemia Time at Explantation (min)** | 46.40 ± 11.70 |
| **Donor Bilirubin (µmol/l)** | 10.65 ± 7.42 | **Cold Ischemia Time (min)** | 401.60 ± 110.39 |
| **Donor ALT (IU/L)** | 59.80 ± 42.76 | **Warm Ischemia Time at Implantation (min)** | 49.20 ± 9.91 |
| **Donor AST (U/L)** | 59.80 ± 42.76 | **Biliary Complications**  **Stenosis** | 3 (20%)  3 |
| **Donor Creatinine (µmol/l)** | 71.51 ± 29.31 |  |  |
| **Donor Sodium (mmol/L)** | 147.20 ± 7.56 |  |  |
