# Supplementary material for: Extrahepatic Bile Duct Organoids as a Model to Study Ischemia/Reperfusion Injury During Liver Transplantation
Source: Transpl Int. 2024 Sep 11;37:13212. doi: 10.3389/ti.2024.13212 (PMC11422091; doi:10.3389/ti.2024.13212)
Supplement: Supplementary file 7 [file Table2.docx]

| **Suppl. Table 2: Antibodies for Histological Staining** | | | | |
| --- | --- | --- | --- | --- |
| Cleaved Caspase 3 (Asp175), Rabbit Polyclonal | Cell Signaling, USA, ref. #9661 | 1:400 | Biotinylated Goat Anti-Rabbit IgG (H+L) RTU, (ref. #ab64256, Abcam, UK), for 15 min | Antigen retrieval with Buffer Citrate pH 6.0 (Zytomed Systems, Germany) for 20min.  Antibody incubation for 1h |
| GGT1/GGT, Mouse Monoclonal [1F9] | Abcam, UK, ref. #ab55138 | 1:200 | Alexa Fluor 488 (Goat anti-Mouse, ref. #A-11001, Invitrogen, USA) (1:100) for 30 min | Antigen retrieval with Buffer Citrate pH 6.0 (Zytomed Systems, Germany) for 20min.  Antibody incubation for 1h |
| Vascular endothelial growth factor A, VEGFA, Mouse Monoclonal (VG-1) | Abcam, UK, ref. #ab1316 | 1:100 | Biotinylated Goat Anti-Mouse IgG (H+L) RTU, ref. #ab64255, Abcam, UK, for 15 min | Antigen retrieval with Buffer Citrate pH 6.0 (Zytomed Systems, Germany) for 20min.  Antibody incubation for 1h |
| Hypoxia-inducible factor 1-alpha, HIF1α, Rabbit Polyclonal | Abcam, UK, ref. #ab51608 | 1:300 | Biotinylated Goat Anti-Rabbit IgG (H+L) RTU, ref. #ab64256, Abcam, UK, for 15 min | Antigen retrieval with Buffer Citrate pH 6.0 (Zytomed Systems, Germany) for 20min.  Antibody incubation for 1h |
| EpCAM, Rabbit Monoclonal, [D9S3P] | Cell Signaling, USA, ref. #14452 | 1:200 | Alexa Fluor 647 (Goat anti-Rabbit, ref. #A-21244, Invitrogen, USA) (1:250) for 30min | Antigen retrieval with Buffer Citrate pH 6.0 (Zytomed Systems, Germany) for 20min.  Antibody incubation for 1h |
| Transcription factor SOX-9 Mouse Monoclonal [3C10] | Abcam, UK, ref. #ab76997 | 1:250 | Alexa Fluor 488 (Goat anti-Mouse, ref. #A-11001, Invitrogen, USA) (1:250) for 30 min | Antigen retrieval with Buffer Citrate pH 6.0 (Zytomed Systems, Germany) for 20min.  Antibody incubation for 1h |
| Cytokeratin 19 Rabbit Monoclonal [EP1580Y] | Abcam, UK, ref. #ab195872 | 1:400 | Alexa Fluor 647 (Goat anti-Rabbit, ref. #A-21244, Invitrogen, USA) (1:250) for 30 min | Antigen retrieval with Buffer Citrate pH 6.0 (Zytomed Systems, Germany) for 20min.  Antibody incubation for 1h |
| Anti-Human Hepatocyte, AHH Mouse Monoclonal [OCH1E5] | Agilent, USA, ref. #M7158 | 1:200 | Alexa Fluor 488 (Goat anti-Mouse, ref. #A-11001, Invitrogen, USA) (1:250) for 30 min | Antigen retrieval with Buffer Citrate pH 6.0 (Zytomed Systems, Germany) for 20min.  Antibody incubation for 1h |
| Tight junction protein 1, TjP1 (Rabbit Polyclonal | Sigma-Aldrich, Germany, ref. #HPA-001636 | 1:150 | Alexa Fluor 488 (Goat anti-Rabbit, ref. #A-11008, Invitrogen, USA) (1:200) for 30 min | Antigen retrieval with Buffer Citrate pH 6.0 (Zytomed Systems, Germany) for 20min.  Antibody incubation for 1h |
| Cyclin 1D, Rabbit monoclonal  [EPR2241] | Abcam, UK, ref. #134175 | 1:100 | Biotinylated Goat Anti-Rabbit IgG (H+L) RTU, ref. #ab64256, Abcam, UK, for 15 min | Antigen retrieval with Buffer Citrate pH 6.0 (Zytomed Systems, Germany) for 20min.  Antibody incubation for 1h |
| Homo sapiens Acyl-CoA synthetase long-chain family member 4, ACSL4 | ACD, Bio-Techne, USA, ref. #408301 | - |  | Target Retrieval buffer (ACD, Bio-Techne, USA) for 15 min. Probe incubation for 2h |
| Homo Sapiens SLC2A1 | ACD, Bio-Techne, USA, ref. #423141 | - |  | Target Retrieval buffer (ACD, Bio-Techne, USA) for 15 min. Probe incubation for 2h |
| Negative Control DapB | ACD, Bio-Techne, USA, ref. #310043 | - |  | Target Retrieval buffer (ACD, Bio-Techne, USA) for 15 min. Probe incubation for 2h |
